# Patient-specific air puff-induced loading using machine learning

**DOI:** 10.3389/fbioe.2023.1277970

**Published:** 2023-11-08

**Authors:** Nada A. Desouky, Mahmoud M. Saafan, Mohamed H. Mansour, Osama M. Maklad

**Affiliations:** ^1^ Mechanical Power Engineering Department, Faculty of Engineering, Mansoura University, Mansoura, Egypt; ^2^ Computers and Control Systems Engineering Department, Faculty of Engineering, Mansoura University, Mansoura, Egypt; ^3^ School of Engineering, Centre for Advanced Manufacturing and Materials, University of Greenwich, London, United Kingdom

**Keywords:** air puff pressure, intraocular pressure (IOP), ocular biomechanics, fluid-structure interaction (FSI), reduced order modelling, machine learning (ML), Gradient Boosting Regressor (GBR)

## Abstract

**Introduction:** The air puff test is a contactless tonometry test used to measure the intraocular pressure and the cornea’s biomechanical properties. Limitations that most challenge the accuracy of the estimation of the corneal material and the intraocular pressure are the strong intercorrelation between the intraocular pressure and the corneal parameters, either the material properties that can change from one person to another because of age or the geometry parameters like central corneal thickness. This influence produces inaccuracies in the corneal deformation parameters while extracting the IOP parametric equation, which can be reduced through the consideration of the patient-specific air puff pressure distribution taking into account the changes in corneal parameters. This air puff pressure loading distribution can be determined precisely from the fluid-structure interaction (FSI) coupling between the air puff and the eye model. However, the computational fluid dynamics simulation of the air puff in the coupling algorithm is a time-consuming model that is impractical to use in clinical practice and large parametric studies.

**Methods:** By using a supervised machine learning algorithm, we predict the time-dependent air puff pressure distribution for different corneal parameters via a parametric study of the corneal deformations and the gradient boosting algorithm.

**Results:** The results confirmed that the algorithm gives the time-dependent air puff pressure distribution with an MAE of 0.0258, an RMSE of 0.0673, and an execution time of 93 s, which is then applied to the finite element model of the eye generating the corresponding corneal deformations taking into account the FSI influence. Using corneal deformations, the response parameters can be extracted and used to produce more accurate algorithms of the intraocular pressure and corneal material stress-strain index (SSI).

**Discussion:** Estimating the distribution of air pressure on the cornea is essential to increase the accuracy of intraocular pressure (IOP) measurements, which serve as valuable indicator of corneal disease. We find that the air puff pressure loading is largely influenced by complex changes in corneal parameters unique to each patient case. With our innovative algorithm, we can preserve the same accuracy developed by the CFD-based FSI model, while reducing the computational time from approximately 101000 s (28 h) to 720 s (12 min), which is about 99.2% reduction in time. This huge improvement in computational cost will lead to significant improvement in the parametric equations for IOP and the Stress-Strain Index (SSI).

## 1 Introduction

Ophthalmology clinical practice utilizes the non-contact air puff tonometry test to measure the human cornea’s biomechanical properties and the Intraocular Pressure (IOP). Precise IOP is an essential aspect in the evaluation of patients at risk for eye diseases such as glaucoma which involves an increase in IOP above normal levels leading to optic nerve damage. Glaucoma is one of the leading asymptomatic causes of permanent blindness in the developed world. A common reason for the IOP increase is the aqueous humuor not draining properly due to blockage of the trabecular meshwork (Stevens et al., 2013). The Ocular Response Analyzer (ORA) (Luce, 2005) was the first tonometry device that used an air puff to determine the ocular biomechanical properties, using a dynamic infrared signal analysis. Then, a new development for better visualisation of the cornea’s deformation was conducted with the aid of the ultra-high-speed Scheimpflug camera using the Corvis-St tonometer (Koprowski, 2014). Corvis-St tonometer applies a concentrated air puff into the centre of the cornea, causing deformation in the cornea’s geometry, which regains its original configuration due to its elasticity. By using image processing, the corneal deformation is recorded to estimate the corneal biomechanical properties and the IOP using a programmed parametric equation.

Another degenerative eye disease of the cornea is keratoconus, where the cornea progressively thins over time, making the shape of the thinned cornea a cone with protrusion (Bao et al., 2016). This is the result of major changes in the thickness, shape, and biomechanical properties (Read and Collins, 2011), which usually produce irregular astigmatism with blurry vision. The tonometry measurements of IOP in patients with keratoconus tend to be lower due to the strong correlation between the IOP measurements and the altered biomechanical properties, particularly the Central corneal thickness (CCT) (Patel and McLaughlin, 1999), (Brooks et al., 1984). Moreover, understanding the biomechanical characteristics and structure of the cornea in keratoconic eyes can also help to clarify the pathophysiology and aetiology of this disorder, which can aid in its treatment (Ambekar et al., 2011).

Therefore, accurate measurements of both the IOP and corneal biomechanical parameters *in vivo* are of utmost importance; however, the challenge is the mutual dependence between the two making it hard to separate the effect of IOP from the biomechanical parameters like thickness and material stiffness on the corneal response parameters (Liu and Roberts, 2005). The solution to the challenge is to solve an inverse problem to assess a more accurate measurement of the corneal material behaviour based on an improved value of the IOP (Maklad et al., 2020a), (Eliasy et al., 2019).

This demonstrates the necessity to study the fluid-structure interaction between the air puff and the cornea (Maklad et al., 2021). So, in order to reduce the association between the IOP and the corneal parameters and consequently increase the IOP estimation’s accuracy, the air puff pressure distribution profiles on the cornea should be taken into account when measuring the corneal deformation (Maklad et al., 2020a). This pressure load exerted on the cornea exhibits dynamically significant changes as a response to the shape of the corneal deformation, which can change clinical interpretations (Yousefi et al., 2022). This effect was called the effect of corneal load alteration with surface shape (CLASS) by Yousefi et al. (Yousefi et al., 2022). As a result, the air puff pressure value and distribution should be obtained based on the patient’s corneal parameters (Maklad et al., 2020b; Maklad et al., 2021; Yousefi et al., 2022).

While the co-simulation of the Fluid-Structure Interaction (FSI) helped to get accurate values of the pressure distribution and the corneal deformation (Simonini and Pandolfi, 2016; Ariza-Gracia et al., 2018; Maklad et al., 2018), the evolution of considering the FSI approach led to a precise estimation of the changing value and distribution of the air jet pressure with the corneal characteristics which led to the biomechanically corrected equations for estimating the accurate IOP and the corneal material stress-strain index (Maklad et al., 2020a), (Maklad et al., 2020b), (Maklad et al., 2018). However, the correct prediction of the air puff pressure in the CFD model for the FSI approach was a time-consuming model, which took too long to get the accurate air puff pressure for each patient-specific geometry.

Our primary goal in this study is to investigate how the corneal characteristics, separately, affect the air puff pressure measurements and produce a new algorithm that uses machine learning to reduce the computational cost. Despite the fact that numerical models of fluid flow have been of significant research interest in many physical and mechanical phenomena, especially when the physical process experiences FSI (Maklad et al., 2020b), (Maklad et al., 2018), (Ariza-Gracia et al., 2015), some recent works (Kutz, 2017; Brunton et al., 2020; Usman et al., 2021) indicated that machine learning (ML) have the potential to be used as a replacement for some of the time-consuming numerical solvers providing reduced-order models, improved optimization performance, and reduced computational cost. Thus, we have established a supervised regression ML algorithm using the Gradient Boosting Regressor (GBR) to estimate the time-dependent air puff corneal pressure distribution profiles to be used as a replacement for the CFD model in the FSI co-simulation of Maklad et al. (Maklad et al., 2020b) to reduce the computational cost and hence update a new version of the algorithms of the IOP and the corneal material estimation. The primary contribution of the current study is to examine the effect of changing the corneal parameters: the IOP, CCT, and material stiffness coefficient (μ) on the air puff pressure profiles, propose a GBR machine learning algorithm for estimating patient-specific air puff pressure loading, and apply the predicted pressure loading to the FE model of the eye in the ABAQUS software to produce the corneal deformation without the need for the CFD time-consuming model.

The organization of the paper is as follows. In Section 2, the processing of the input dataset with the proposed regression algorithm and methods of its evaluation are introduced. Section 3 presents the results of the GBR fitted algorithm, investigating the effect of each corneal parameter on the estimation of the air puff corneal pressure load, with a graphical user interface of the GBR algorithm. Moreover, the predicted corneal deformation based on the predicted air puff pressure, with a full validation of the algorithm with the clinical data is presented. Section 4 concludes the paper and introduces our future work.

## 2 Materials and methods

**FIGURE 1:**
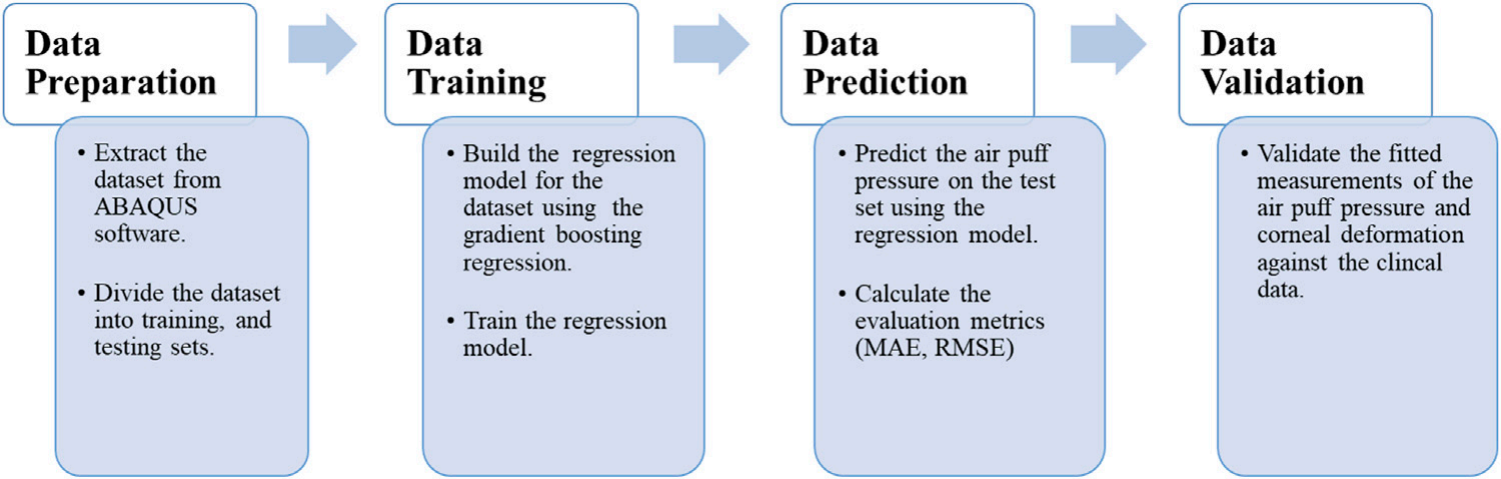
Flowchart of the data processing in the current study.

The current section begins by presenting the data set used in our approach and the algorithm used in the learning and predicting process. Then, we introduce the evaluation matrices used to evaluate our model’s performance, and the section ends by constructing a validation study between the clinical data extracted from patients’ cases and our current algorithm. Figure 1 depicts the data processing steps in our algorithm, which will be described in detail in the following sub-sections.

### 2.1 Data collection and processing

**FIGURE 2:**
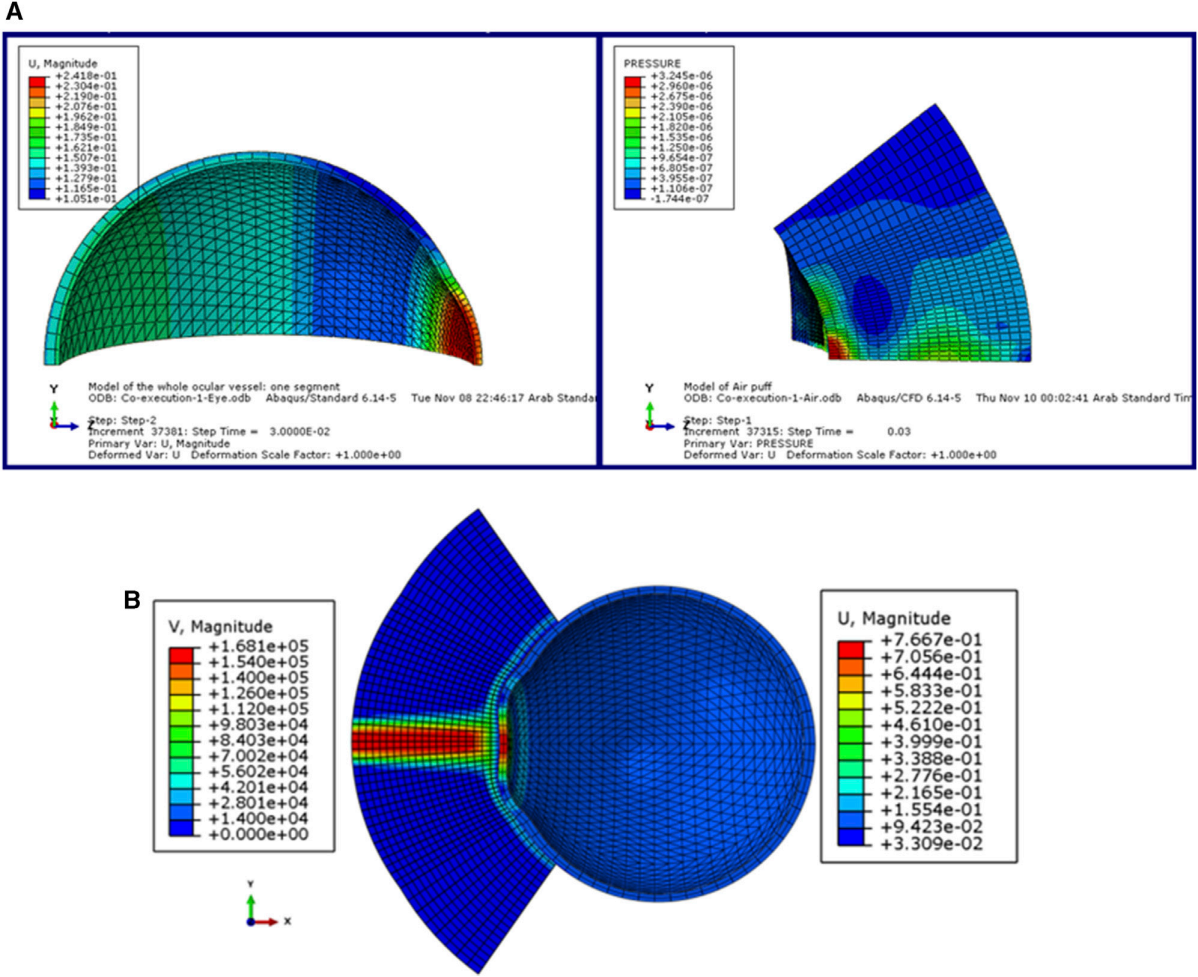
**(A)** The model of the eye and the air puff with the deformation and pressure values respectively, and **(B)** The FSI coupled model of the air puff test in ABAQUS software (Maklad, 2019).

In this section, we explain the input dataset of our algorithm of the fully coupled FSI simulation of the air jet CFD model and the FE model of the eye obtained from the model of (Maklad et al., 2020b). Figure 2 shows the coupled FSI model from the ABAQUS 6.14 software. Due to the rotational symmetry of the results of the parametric study of (Maklad et al., 2020b) in both domains, a quarter of the two domains were simulated to save running time as shown in Figure 2A, while Figure 2B shows the deformation values of the whole ocular vessel with the air puff’s velocity values shown in (mm/s) on the left, while the eye model’s deformation values are shown in (mm) on the right.

The parameters included in the study are the IOP, the CCT, and the material stiffness coefficient (μ). The selection of the six values of the material stiffness coefficient is based on the relation with age as obtained by (Elsheikh et al., 2010) with μ = 0.0328 representing age = 30 years and μ = 0.1082 representing age = 100 years. The influence of each parameter on the estimation of the pressure distribution on the cornea is studied while the other parameters are fixed. First, the pressure distribution on the cornea is estimated using the range of the IOP values from 10 to 25 mmHg at CCT of 445 μm, and material stiffness coefficient of 0.0541. Then, at IOP = 15 mmHg and material stiffness coefficient = 0.0541, the influence of different values of the CCT in the range from 445 to 645 µm is tested on the calculation of the pressure on the cornea. Likewise, the pressure distribution on the cornea is estimated against the variations of the material stiffness coefficient in the range from 0.0328 to 0.1082 at the same IOP of 15 mmHg and the CCT of 545 µm. Finally, to estimate and evaluate the pressure distribution on the cornea for different corneal parameters, a complete simulation of 17 different new cases of the FSI model of Maklad et al. (Maklad et al., 2020b) in the ABAQUS 6–14 software has been performed. Table 1 summarises the corneal parameters that have been included in the study.

**TABLE 1:** The values of the corneal parameters used to test our regression model.

IOP (mmHg)	10	13	15	17	20	22	24	25
CCT (µm)	445	495	545	595	645			
μ	0.0328	0.0541	0.0683	0.0811	0.1082			

### 2.2 The Gradient Boosting Regressor (GBR) algorithm

GBM or Gradient Boosting Machine is a popular machine learning algorithm used for both regression and classification tasks. Using the GBM algorithms to solve regression problems is called the Gradient Boosting Regressor (GBR) technique, which is based mainly on the loss function, the base learner, and the additive model (Friedman, 2001). GBR ensemble model contains a series of tree models arranged sequentially, allowing each subsequent model to learn from the errors of its predecessor, and the predictions are generated by boosting the weak models, typically decision trees, to create a more powerful and robust predictive model (Friedman et al., 2000). To implement the GBR algorithm, it is necessary to specify the hyperparameters, which are an integral part of the learning algorithm and significantly affect its performance and accuracy. The parameters used in our algorithm are the squared-loss function, a learning rate of 0.3, 2,700 boosting stages, max_depth of 6, and the min_samples_split of 5. In gradient descent, the loss function is optimized for model generalization and the default option is the squared error that defines the calculated error as the residual.

Another crucial parameter is the learning rate, which indicates the rate at which the contribution of each tree shrinks. Careful selection of the learning rate is vital; a lower choice results in a slower learning process but increases the reliability and efficiency of the model. The number of estimators’ parameters represents the number of boosting stages to execute, and the higher the number, the better the performance. Then, there is the max-depth parameter, which constrains the nodes in each tree, and the final parameter is the minimum number of samples needed to split an internal node (sklearn.ensemble.GradientBoostingRegressor, 2020). In each subsequent run of the algorithm, the dataset was randomly split into 70% for training and 20% for testing, with the remaining data allocated for validation. Simulations were performed on an Intel Core i7 8550U processor with 8 GB RAM. A detailed discussion of the mathematical formulation of GBR models will follow.

For an additive model with a given input $x_{i}$ and prediction ${\hat{y}}_{i}$ , the form of the model of GBR is of the following form (sklearn.ensemble.GradientBoostingRegressor, 2020):

$${\hat{y}}_{i}=F_{M}\left(x_{i}\right)=\sum_{m=1}^{M}\vartheta h_{m}\left(x_{i}\right)$$ (1)

The constant M denotes the n_estimators parameter, ϑ is the learning rate parameter, and $h_{m}$ is the basic function that is known as the weak learner’s estimator. As is known, the GBR algorithm is built in a greedy fashion (sklearn.ensemble.GradientBoostingRegressor, 2020):

$$F_{m}\left(x\right)=F_{m-1}\left(x\right)+\vartheta h_{m}\left(x\right)$$ (2)

The newly inserted tree $h_{m}$ is fitted at each iteration to reduce a sum of losses $L_{m}$ , given the previous ensemble $F_{m-1}$ :

$$h_{m}=\arg\min_{h}L_{m}=\arg\min_{h}\sum_{i=1}^{n}l\left(y_{i},F_{m-1}\left(x_{i}\right)+h\left(x_{i}\right)\right)$$ (3)

As a result, with the loss parameter $l\left(y_{i},F\left(x_{i}\right)\right)$ , the model formula is now:

$$F_{m}\left(x\right)=F_{m-1}\left(x\right)+\vartheta \;\arg\min_{h}\sum_{i=1}^{n}l\left(y_{i},F_{m-1}\left(x_{i}\right)+h\left(x_{i}\right)\right)$$ (4)

By default, for the least-squares loss, the initial model $F_{0}$ is selected as the mean of the target values, which is the constant that minimizes the loss. So, with a first-order Taylor approximation, the value of l can be approximated as follows (sklearn.ensemble.GradientBoostingRegressor, 2020):

$$l\left(y_{i},F_{m-1}\left(x_{i}\right)+h\left(x_{i}\right)\right)\approx l\left(y_{i},F_{m-1}\left(x_{i}\right)\right)+h\left(x_{i}\right){\left[\frac{\partial l\left(y_{i},F\left(x_{i}\right)\right)}{\partial F\left(x_{i}\right)}\right]}_{F=F_{m-1}}$$ (5)

The quantity $-{\left[\frac{\partial l\left(y_{i},F\left(x_{i}\right)\right)}{\partial F\left(x_{i}\right)}\right]}_{F=F_{m-1}}$ represents the negative gradient $-g_{i}$ , which is calculated using a gradient descent method, and by removing the constant terms, it approximately results in:

$$h_{m}\approx \arg\min_{h}\sum_{i=1}^{n}h\left(x_{i}\right)g_{i}$$ (6)

The gradients are updated at each iteration until convergence is achieved and this can be considered some kind of gradient descent in a functional space (sklearn.ensemble.GradientBoostingRegressor, 2020).

To assess the effectiveness of the GBR algorithm, it is essential to quantify the model error. We used the Mean Absolute Error (MAE) and Root Mean Square Error (RMSE) criteria to measure the error in our approach. The MAE provides insight into the difference between observed and actual data, calculated mathematically as follows:

$$\text{MAE}=\sum_{i=1}^{n}\frac{\left|\text{Predicted}-\text{Actual}\right|}{n}$$ (7)

By calculating the standard deviation of the prediction errors, the RMSE can be estimated, which is represented as:

$$\text{RMSE}=\sqrt{\sum_{i=1}^{n}\frac{{\left(\text{Predicted}-\text{Actual}\right)}^{2}}{n}}$$ (8)

Additionally, as the computational cost is our research concern, another significant consideration is the computational time which represents the time the model has taken to learn and produce predictions from the input data.

### 2.3 Validation of the GBR algorithm

To validate our algorithm against the literature, the normalised air puff pressure was compared to the studies by Kling et al. (Kling et al., 2014) and Muench et al. (Muench et al., 2019) at two different time steps at T = 10 and 16 m. Then, to clinically validate our algorithm, a set of clinical data with a wide range of corneal parameters for four healthy patients provided by Vincieye Clinic in Milan, Italy, and Rio de Janeiro Corneal Tomography and Biomechanics Study Group, Brazil, was selected to be the input data for our GBR algorithm to predict their air puff loading and compare the corneal deformations. The ethical standards set out in the 1964 Declaration of Helsinki and their revision in 2013 were observed and all patients provided written informed consent before using their de-identified data in research. The selected clinical cases are summarised in Table 2.

**TABLE 2:** The corneal parameters of the four clinical cases used in the validation.

Corneal parameter/Case id	Case 1 (Age = 73)	Case 2 (Age = 54)	Case 3 (Age = 63)	Case 4 (Age = 40)
IOP (mmHg)	17.5	18	15	24
CCT (µm)	560	579	548	582
μ	0.061	0.054	0.057	0.051

**FIGURE 3:**
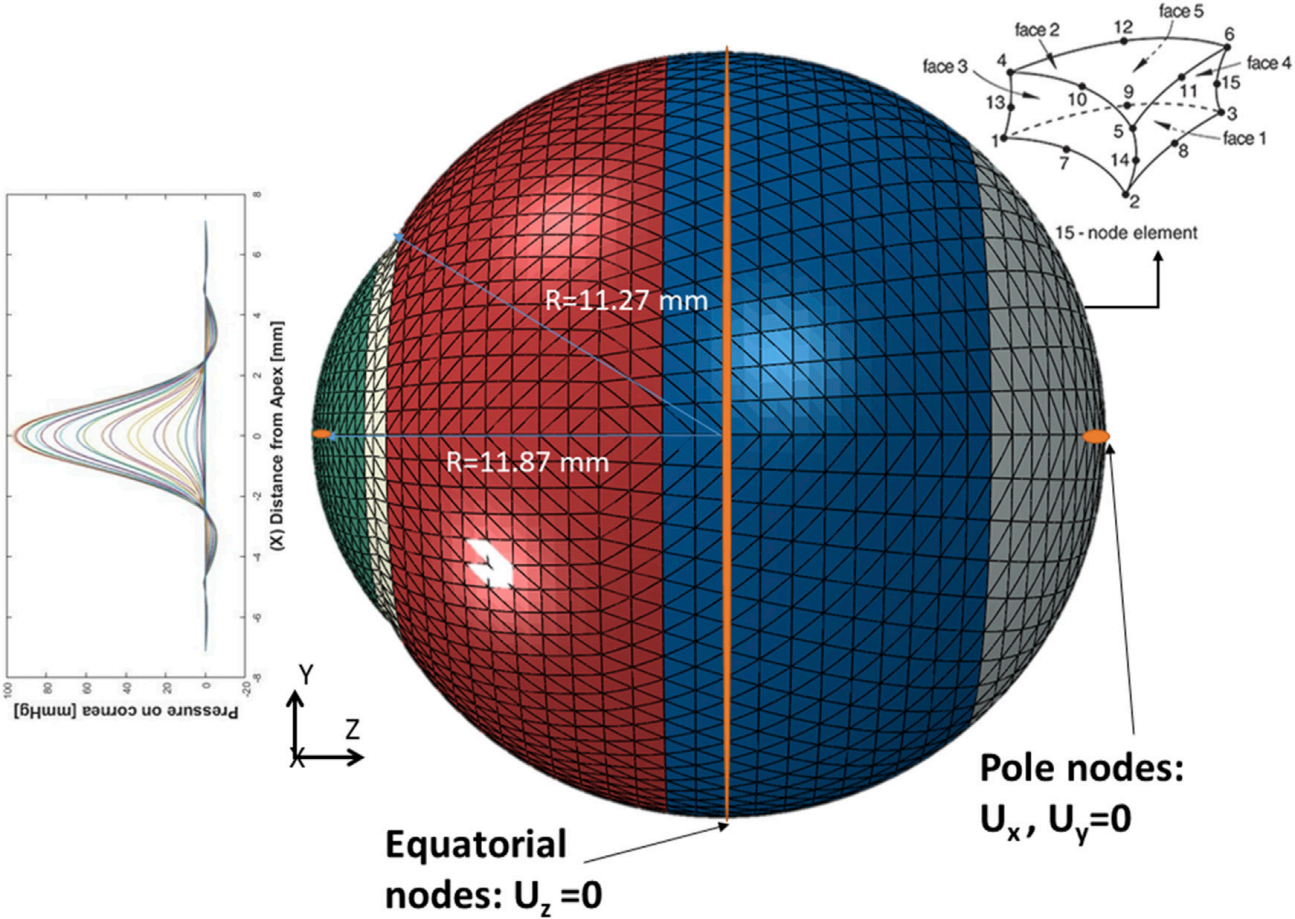
The Finite Element model of the eye along with the applied boundary conditions.

After predicting the air puff pressure loading with the GBR algorithm, we applied it to the FE model of the eye in ABAQUS 6–14. The three-dimensional eye FE model consisted of 10,000 fifteen-nodded continuum elements (C3D15H) with nine integration points, arranged in two layers, and distributed along 15 rings in the cornea and 35 rings in the sclera. The rigid body motion of the FE model of the eye was prevented in the Z-direction at the equatorial nodes, while the motions in the *X* and *Y* directions of the posterior and anterior pole nodes were restricted with free movement in the Z-direction, as shown in Figure 3, which is the same as applied in (Maklad et al., 2020a), (Eliasy et al., 2019), (Maklad, 2019).

## 3 Results and discussion

After building the GBR model, we used it to obtain the pressure distribution on the cornea at different time steps of the test for the corresponding patent-specific corneal parameters: IOP, CCT, and material stiffness coefficient (μ). A graphical user interface of the GBR algorithm was built in MATLAB to make it easier to change the corneal parameters and generate new predictions of the air puff pressure distribution.

### 3.1 Effect of the corneal parameters (IOP, CCT, and μ)

**FIGURE 4:**
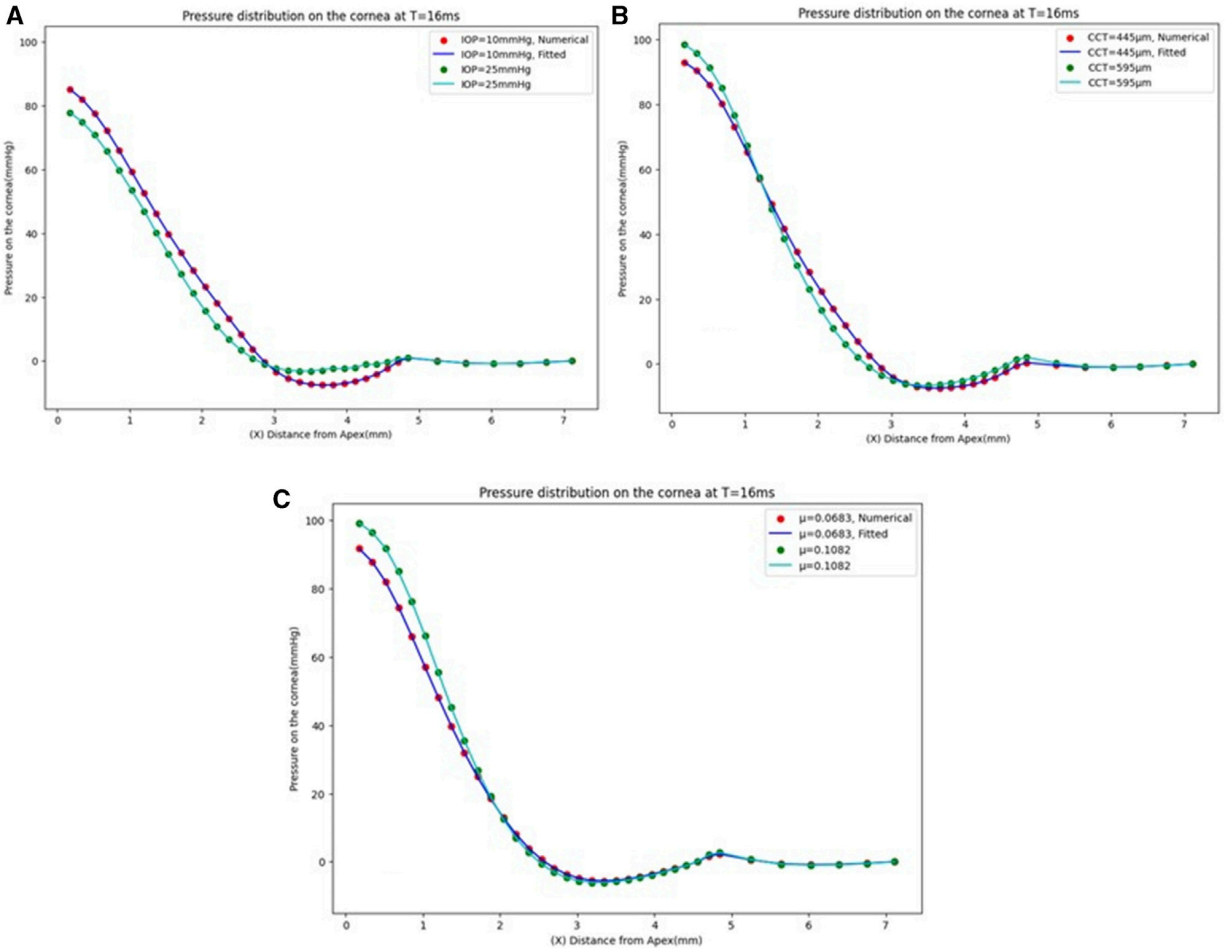
Pressure distribution on the cornea at T = 16 m. **(A)** At CCT = 445 µm and µ = 0.0541, **(B)** At IOP = 15 mmHg and µ = 0.0541, and **(C)** At IOP = 15 mmHg and CCT = 545 µm.

The GBR model has been analysed to see the effect of changing the IOP on the pressure distribution estimation. The pressure distribution on the cornea is obtained at the corneal surface for eight different values of the IOP (10, 13, 15, 17, 20, 22, 24, and 25 mmHg), while the other parameters are the same at a CCT of 445 µm and a material stiffness coefficient of 0.0541. The results of the comparison between the fitted GBR algorithm and the numerical values obtained from the FSI model show a good agreement with MAE = 0.0212, RMSE = 0.0682, and an execution time of 12 s. Then, we demonstrated the influence of different values of the CCT in the range (445, 495, 545, 595, and 645 µm) on the pressure distribution on the cornea at an IOP of 15 mmHg and a material stiffness coefficient of 0.0541, and the fitted algorithm shows high agreement with the numerical ABAQUS model with MAE = 0.0171, RMSE = 0.0578, and an execution time of 10 s. Finally, the variations of the material stiffness coefficient in the range (0.0328, 0.0541, 0.0683, 0.0811, and 0.1082) to represent the age effect are tested at IOP of 15 mmHg and CCT of 545 μm, and the fitted pressure distribution agrees well with the numerical ABAQUS model with an MAE of 0.0113, an RMSE of 0.0491, and an execution time of 12 s. Figure 4 shows the effect of changing each corneal parameter separately on the fitted pressure load at T = 16 m. The plots for minimum and maximum values for the parameters are shown to feel the influence of changing the corneal parameters on the predicted pressure load. They show how the algorithm predictions agreed very much with the numerical values obtained from the FSI model from the ABAQUS software, and Table 3 shows the RMSE estimated between them for all cases at T = 16 m. It is clear from the comparison how the maximum value for the pressure at the apex is different between the two models. Moreover, the difference is not only in the maximum value of the pressure at the apex but also is in the pressure distribution, and this is what we want the ML algorithm to learn and apply to the different models with patient-specific corneal parameters.

**TABLE 3:** The RMSE estimated the pressure load by changing each corneal parameter separately.

Corneal parameter		RMSE
IOP (mmHg)	10	0.0186
	13	0.0453
	15	0.0511
	17	0.0530
	20	0.0769
	25	0.0894
CCT (µm)	445	0.0322
	495	0.1021
	545	0.0467
	595	0.0458
	645	0.0759
µ	0.0328	0.0201
	0.0541	0.0340
	0.0683	0.0119
	0.0811	0.0362
	0.1082	0.0533

### 3.2 Estimation of the air pressure distribution on the cornea

**FIGURE 5:**
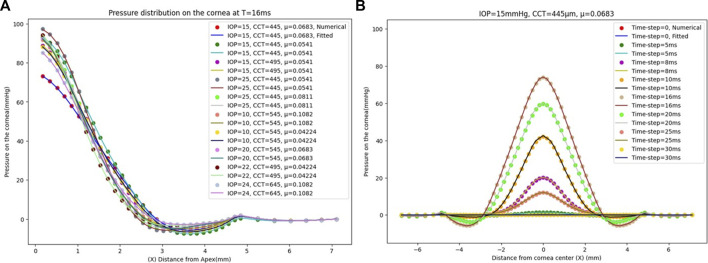
**(A)** The comparison between the numerical ABAQUS model and the fitted GBR algorithm of the pressure distribution on the cornea at T = 16 m with a different corneal parameter of IOP, CCT, and μ. **(B)** The pressure distribution on the cornea at different time steps for IOP = 15 mmHg, CCT = 445 µm, and µ = 0.0683.

We analysed the effect of considering all the previous corneal parameters combined on the pressure loading and tested it with the numerical data model to evaluate its effectiveness. To build our model, 17 new different cases were simulated on ABAQUS 6–14 to get their results as the input dataset to our algorithm. The results of the pressure profile in Figure 5A show that changing one parameter of the corneal parameters can change the whole pressure profile and Figure 5B shows the pressure distribution on the cornea at the different time steps. The fitted algorithm with the numerical data model shows a good agreement with an MAE of 0.0258, an RMSE of 0.0673, and an execution time of 93 s, and the RMSE estimated between them for each case is calculated and provided in Supplementary Table S1 in the Supplementary Material. The findings show that there is no systematic relationship between changing the air puff pressure loading with each parameter separately because the pressure distribution on the cornea is affected by the other parameters, all of which must be considered to obtain an accurate air puff pressure.

### 3.3 Validation of the GBR algorithm

**FIGURE 6:**
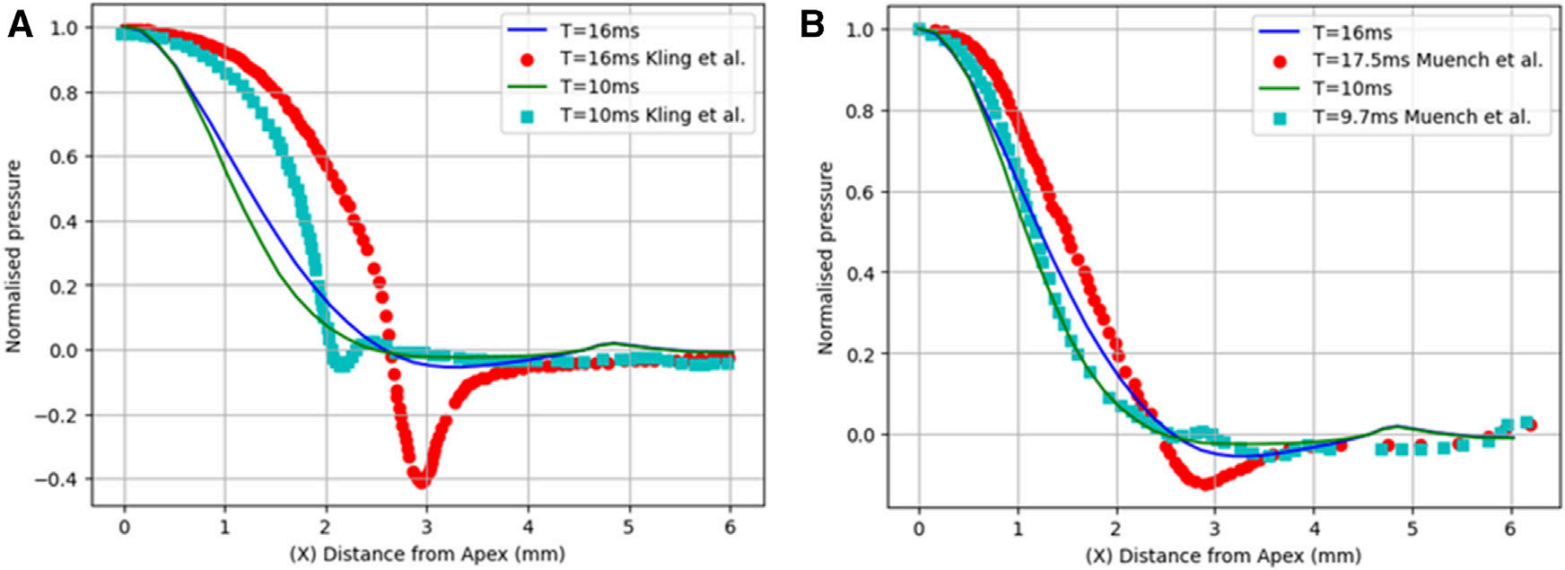
Comparison of the normalized pressure distribution with two studies from previous literature: **(A)** Kling et al. (Kling et al., 2014) and **(B)** Muench et al. (Muench et al., 2019).

First, the normalised air puff pressure was compared to that in the studies by Kling et al. (Kling et al., 2014) and Muench et al. (Muench et al., 2019) at two different time steps at T = 10 and 16 m with an acceptable agreement as shown in Figure 6. From this comparison, we believe that the distribution reported by Muench et al. (Muench et al., 2019) is closer to reality since it takes the fluid-structure interaction into account. However, the distribution reported by Kling et al. (Kling et al., 2014) is based on a completely rigid cornea which does not take the FSI influence into account as we have proven that the air puff pressure distribution is significantly affected by the corneal biomechanical parameters.

**FIGURE 7:**
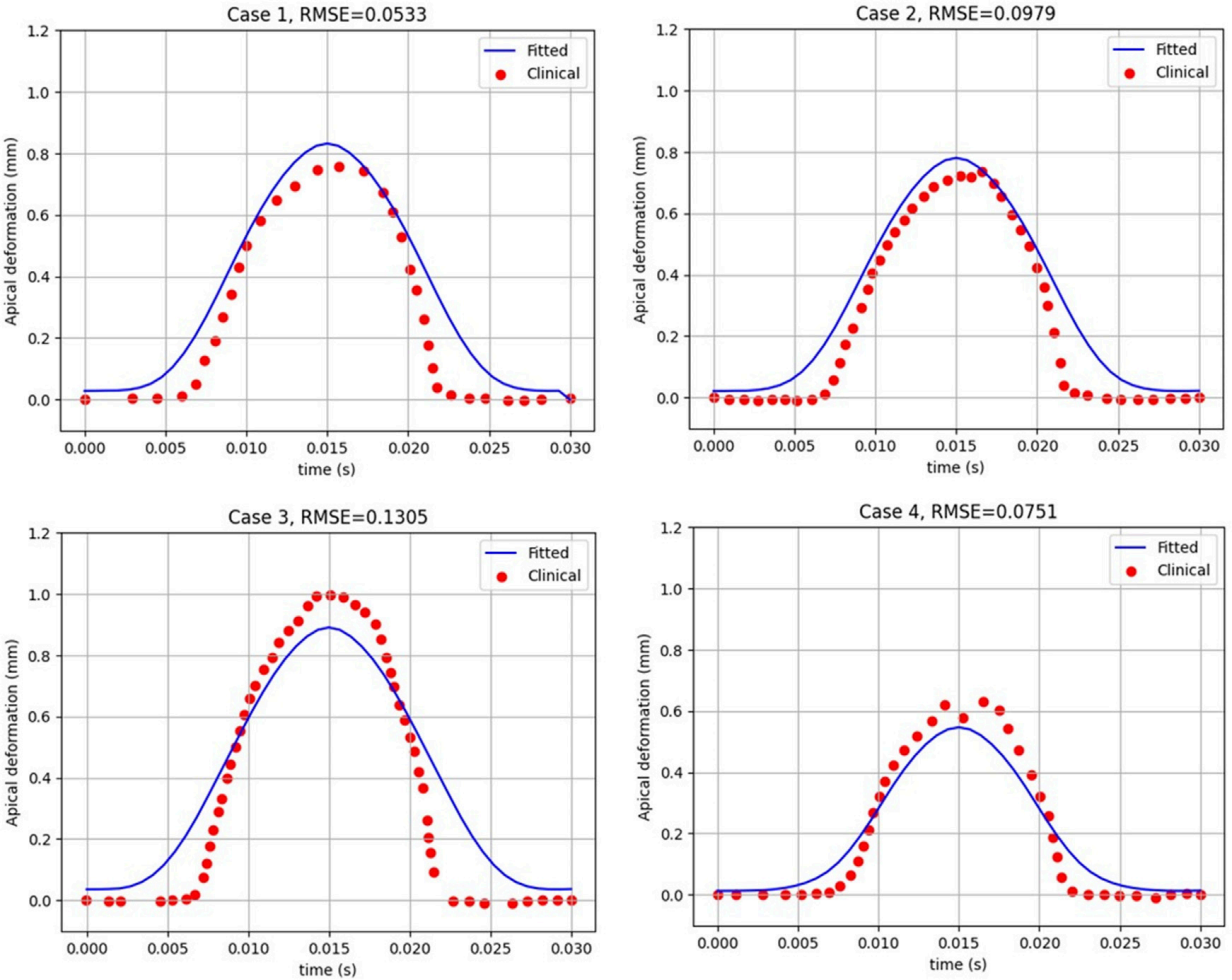
The apical deformation resulting from the GBR + FE algorithm compared against its clinical data reference for four clinical cases.

**FIGURE 8:**
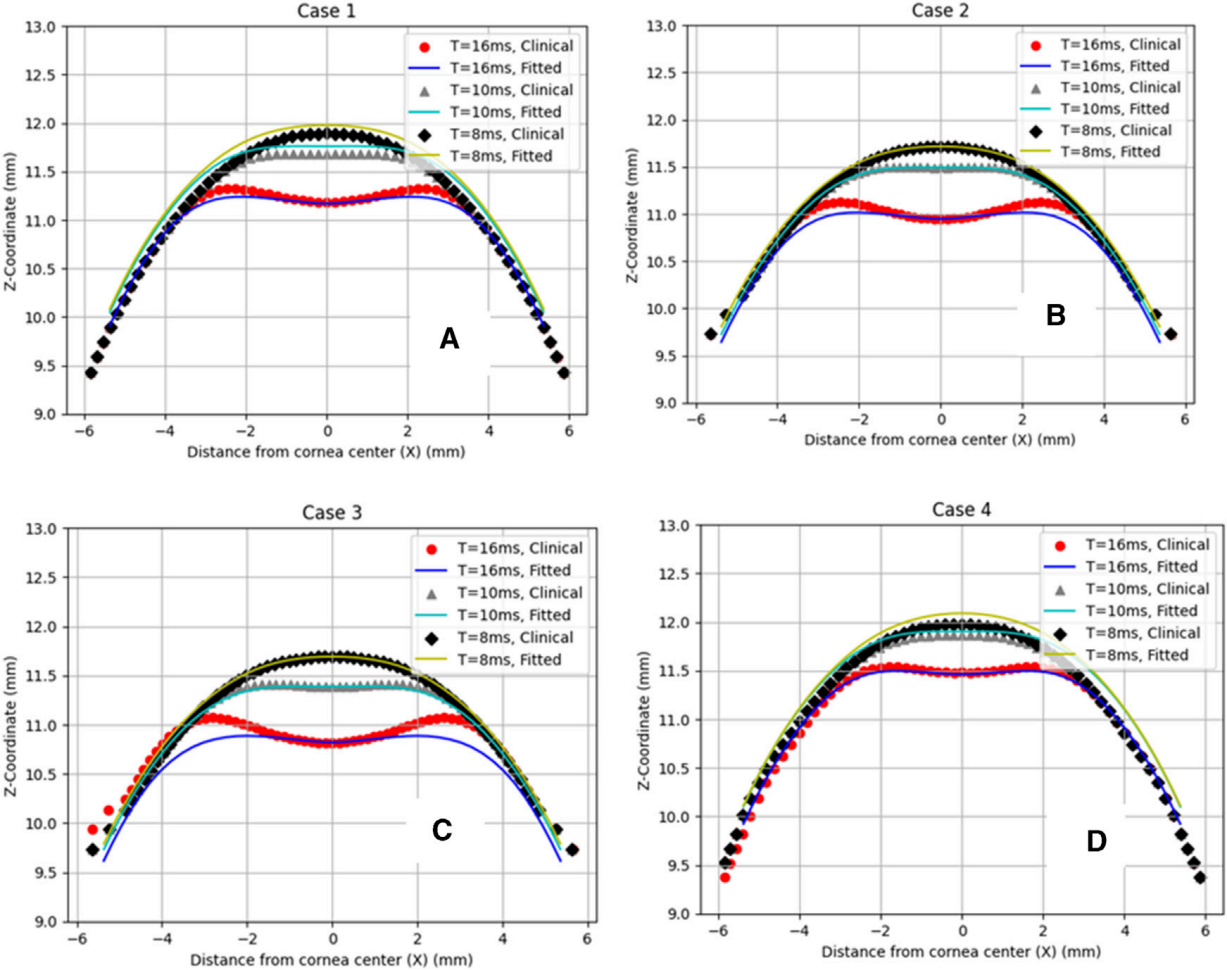
Comparison of the corneal deformation results from the fitted algorithm with the four clinical cases. **(A)** Case 1: IOP = 17.5 mmHg, CCT = 560 µm, µ = 0.061, **(B)** Case 2: IOP = 18 mmHg, CCT = 579 µm, µ = 0.054, **(C)** Case 3: IOP = 15 mmHg, CCT = 548 µm, µ = 0.057, **(D)** Case 4: IOP = 24 mmHg, CCT = 582 µm, µ = 0.051.

Then, to clinically validate our algorithm, a set of clinical data with a wide range of corneal parameters for four healthy patients was selected. After predicting the air puff pressure loading with the GBR algorithm, we applied it to the FE model of the eye in ABAQUS 6–14. The FE model took 10 min to finish and generate the corneal deformation from the predicted air puff pressure loading, and these deformations were then compared with the clinical deformation to complete the full validation of our algorithm and be used as an alternative approach to the CFD model to reduce its computational time within minutes (720 s = 12 min) instead of waiting many hours in the FSI model (101,000 s = 28 h), which is approximately 99.2% reduction in time. Figure 7 shows the comparison of the temporal profile of the apical deformation with the clinical cases and Figure 8 presents the comparison of the spatial corneal deformation based on the predicted air puff pressure loading with their clinical data, and this comparison shows that this algorithm can produce a good and close behaviour to the real corneal behaviour. Additionally, to show the improvement caused by using the GBR algorithm with the FE model instead of using the FE model only to predict the apical deformation, the RMSE calculated for both cases was compared and presented in Table 4.

**TABLE 4:** The RMSE estimated between the clinical cases with the GBR + FE model and the numerical FE model only for the apical deformation (Maklad, 2019).

Clinical case	RMSE (GBR + FE model vs. clinical)	RMSE (FE model only vs. clinical)
Case 1	0.0533	0.6393
Case 2	0.0979	0.3655
Case 3	0.1305	0.4263
Case 4	0.0751	0.4403

There is a hysteresis effect that causes some delay for the cornea to return back to the original geometry due to the visco-elastic material behaviour of the cornea in clinical cases. This effect is not considered in the material model of the eye in the ABAQUS co-simulation data set, which can cause some changes in the comparison between the clinical and fitted data after the air puff hits the cornea. Moreover, the fatty tissue surrounding the eye and the shooting angle of the air puff have an influence on the induced loading which was not applied in our model.

### 3.4 The graphical user interface of the GBR algorithm

**FIGURE 9:**
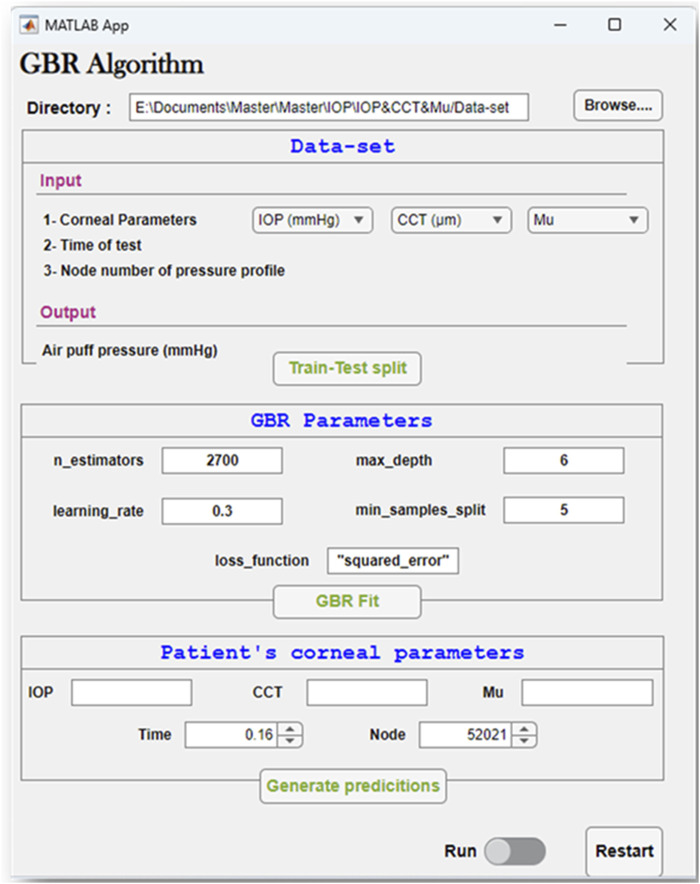
Graphical user interface of the GBR algorithm to estimate the air puff pressure.

To easily manage the interaction between the steps of the GBR algorithm to conduct our study effectively, a graphical user interface was built. Figure 9 depicts the GBR algorithm’s main sections, which are summarized as follows: The input file is imported from its directory, which contains the input data and their labelled outputs. The input data set includes the corneal parameters: the IOP, CCT, and μ, with the time of the test, and the node number of the air puff pressure profile. Their labelled output, which is our desired outcome, is the air puff pressure load. This data set is divided into training and testing sets with a test size of 0.25 and a random state of 50. The hyper-parameters used in our model are shown in Figure 9, which are then used to fit our algorithm by improving the weak learner. Then, the patient-specific corneal parameters are inserted to generate their predictions within up to 2 min.

## 4 Conclusion

Estimating the distribution of air pressure on the cornea is essential to increasing the accuracy of intraocular pressure (IOP) measurements, which serve as valuable indicators of corneal disease. The accuracy of these measurements directly affects the accuracy of the evaluation of the corneal material. While the Fluid-Structure Interaction (FSI) method has successfully provided accurate corneal compressive load predictions based on corneal deformation, it is a time-consuming model that takes too many hours (nearly 28 h) to produce results. In this study, we developed a supervised regression ML algorithm aimed at estimating corneal pressure distribution from corneal parameters while optimizing both accuracy and computational time. The primary result of our research is the creation of a more practical algorithm, namely, the Gradient Boosting Regressor (GBR) model, capable of predicting corneal pressure load considering the influence of the corneal parameters; IOP, central corneal thickness (CCT), material coefficient (representing patient’s age), and the test time step. Our findings show that the air puff pressure loading is largely influenced by complex changes in corneal parameters unique to each patient case. Moreover, this innovative algorithm significantly reduces the computational time compared to the CFD-based FSI approach from approximately 101,000 s (28 h) to 720 s (12 min), which is approximately 99.2% reduction in time, while preserving the same accuracy developed by the FSI algorithm. This huge improvement in computational cost will lead to significant improvement in the parametric equation for IOP and the Stress-Strain Index (SSI) by considering a larger number of full-eye patient-specific models with dynamic topography, which is our plan for further research. These algorithms hold great promise for clinical tonometry measurements due to their accuracy and efficiency.

## Figures and Tables

**Table:** 

**IOP**	Intraocular Pressure
**CCT**	Central Corneal Thickness
**FE**	Finite Element
**CFD**	Computational Fluid Dynamics
**FSI**	Fluid-Structure Interaction
**ORA**	Ocular Response Analyser
**CorVis-ST**	Corneal Visualisation Scheimpflug Technology
**ALE**	Arbitrary Lagrangian-Eulerian
**ML**	Machine Learning
**GBM**	Gradient Boosting Machines
**GBR**	Gradient Boosting Regressor

## Data Availability

The original contributions presented in the study are included in the article/Supplementary Material, further inquiries can be directed to the corresponding author.
