# Supplementary material for: Patient-specific air puff-induced loading using machine learning
Source: Front Bioeng Biotechnol. 2023 Nov 8;11:1277970. doi: 10.3389/fbioe.2023.1277970 (PMC10663333; doi:10.3389/fbioe.2023.1277970)
Supplement: Supplementary file 1 [file DataSheet1.docx]

| Corneal parameter | | | RMSE |
| --- | --- | --- | --- |
| IOP (mmHg) | CCT(μm) | μ |  |
| 10 | 545 | 0.1082 | 0.064979 |
| 10 | 545 | 0.04224 | 0.037123 |
| 10 | 645 | 0.1082 | 0.053441 |
| 15 | 445 | 0.0683 | 0.071868 |
| 15 | 445 | 0.0541 | 0.099724 |
| 15 | 495 | 0.0541 | 0.07102 |
| 15 | 595 | 0.0541 | 0.022063 |
| 15 | 645 | 0.0683 | 0.035776 |
| 15 | 645 | 0.04224 | 0.05616 |
| 20 | 545 | 0.0811 | 0.05591 |
| 20 | 545 | 0.0683 | 0.052987 |
| 20 | 645 | 0.0541 | 0.034699 |
| 20 | 645 | 0.0683 | 0.104353 |
| 22 | 495 | 0.04224 | 0.074413 |
| 22 | 645 | 0.1082 | 0.083329 |
| 24 | 645 | 0.1082 | 0.085027 |
| 24 | 495 | 0.1082 | 0.079132 |
| 25 | 645 | 0.0811 | 0.091387 |
| 25 | 645 | 0.1082 | 0.058659 |
| 25 | 445 | 0.0541 | 0.126839 |
| 25 | 445 | 0.0811 | 0.076591 |
| 25 | 545 | 0.1082 | 0.035683 |
| 25 | 545 | 0.04224 | 0.084109 |
| 25 | 545 | 0.0683 | 0.206953 |

. **Table** S1 The RMSE estimated for the pressure load by changing the whole corneal parameters.

| **The Algorithm**   1. Write an excel file with the input data of the patient-specific corneal parameters to be the test set, which is the target to predict their pressure and deformation values. 2. Run Python code building a Gradient Boosting Regressor (GBR) model learning with the training given patients’ data. 3. With the input data of the test sub-set, predict their corresponding resulted pressure values by the pre-learned model. 4. Use the predicted pressure values as an input file to the FE model at Abaqus. 5. Make a node-set of the deformation nodes of the cornea and run the FE model at Abaqus program. 6. Write the corneal deformations resulting from the deformation nodes with the Python code to a file. |
| --- |
|  |
|  |

**The Code**

import pandas as pd

import matplotlib.pyplot as plt

from sklearn import ensemble

from sklearn.ensemble import GradientBoostingRegressor

#Read the training data’s file

pres1 = pd.read_excel(r'E:\Documents\Master\Master\IOP\IOP&CCT&M/train-data.xlsx')

df1 = pd.DataFrame(pres1)

#Read the test data’s file

pres2 = pd.read_excel(r'C:\Users\laptop home\Downloads\Clinical cases-20230302T113502Z-001\FE\test-data.xlsx')

df2 = pd.DataFrame(pres2)

#The training data’s input, which is the time step, the node number on the corneal profile, the intraocular pressure(IOP), the central corneal thickness (CCT), and material stiffness (Mu).

xdata=df1.loc[:,["time","x","iop","cct","m"]]

#The training data’s output, which is the pressure distribution on the cornea.

ydata=df1["pressure"]

#The test data’s input, and the target is to predict its corresponding pressure values.

x_pred=df2.loc[:,["time","x","iop","cct","m"]]

# Hyperparameters for GradientBoostingRegressor

gbr_params = {

    "n_estimators": 2700,

    "max_depth": 6,

    "min_samples_split": 5,

    "learning_rate": 0.3,

    "loss": "squared_error",

}

# Create an instance of gradient boosting regressor

gbr = ensemble.GradientBoostingRegressor(**gbr_params)

# Fit the model

gbr.fit(xdata, ydata)

#Generate predicitions

pred_test= gbr.predict(x_pred)

#Plot the model

plt.scatter(x_pred["time"], pred_test, color="blue", label="Fitted")

plt.xlabel("time (s)")

plt.ylabel("Pressure (mmHg)")

plt.grid()

plt.legend()

plt.show()
